# Round the Hospitals

**Published:** 1918-06-01

**Authors:** 


### ROUND THE HOSPITALS.

We refer our readers to page 194, where they will find the latest, and we hope the final announcements in regard to the College of Nursing Conference to be held next week. It should be remembered that the Thursday evening and Friday afternoon sessions are open to all those interested in the nursing profession, while the session on Friday morning will be private and confined to past and present matrons who are members of the College. Any nurses or others desiring hospitality for the night of June 6 should communicate at once with Miss Girdlestone, the College of Nursing (Limited by Guarantee), 6 Vere Street, London, W. 1, in order that the necessary arrangements may be made. The two public meetings of the Conference—namely, that of Session I. on Thursday evening, June 6, at 7.30 P.M., and that of Session III. on the afternoon of Friday, June 7, at 3 P.M.—have each a very full programme. Miss Gullan, sister-tutor of St. Thomas's Hospital, will read a paper on "The Higher Education of Nurses in its Relation to Applicants for Training," a subject of the greatest possible importance to all women who aspire to qualify for the nursing profession and to obtain their certificates under the best auspices, which should prove fruitful in results and materially aid their continuous rise to high positions in the field of nursing.

Intense anxiety is being experienced by the relatives and friends of nurses in France consequent upon the outrage committed by the enemy in bombing a wide area covered by hospitals on the night of May 19. "Quite the most horrible thing they have done yet" is the universal verdict. Like the murder of Miss Cavell, this cold-blooded and deliberate slaughter of the helpless rouses the whole world to scorn and indignation. The hospital area chosen for the scene of this tragedy is probably well known to all our readers who have worked in France and Flanders. No spot could have been selected likely to bear richer fruits to hatred and revenge. The calculated barbarity shown by the assailants leaves such a blot upon human nature as can only be eclipsed in contemplation of the superb bearing of our women through those hours of long-drawn-out agony. For them shelter and safety lay close at hand, but not one moved to avail herself of it. Calm and magnificent in self-control, they ministered to the helpless sufferers they could not protect, and many died in the act. We await with some impatience the list of killed and wounded which official reticence still denies. We look, too, for the sculptor who, with sure inspiration, shall crystallise for all time the heroism of that group of sisters in a monument worthy of them and of the Empire. Surely countless generations of young probationers, irked with a discipline whose inner purport they cannot grasp, will learn by such examples to what heights the sublime mastery of self can lead under a system which has succeeded in subordinating natural impulses to a high sense of duty.

South Africa is following the example of other countries in establishing a Voluntary Aid Detachment, and the rate of enrolment is already very satisfactory. The South African Hospital in Richmond Park employs forty-five V.A.D. helpers, and South African residents in London connected by many links with the sisters and patients naturally desire to share in the work. The volume of work already accomplished by South Africans through the many-sided Comforts Committee which ministers to the South African Forces has added very considerably to the efficiency of the corps, and has relieved both officers and men of many discomforts they would have experienced as strangers in the capital. London in these days is more cosmopolitan than ever, and it is charming to observe the centres of strong "Colonial" (if we dare use the word) sentiment, each with its characteristic flavour, which are scattered here and there throughout the town, all vowed to one purpose, the relief of the Mother Country's burden.

Meantime the home supply of V.A.D. helpers still runs short. There seems to be a doubt whether, in spite of all that has been done to publish revised regulations, the members of detachments do yet realise that full-time workers in auxiliary hospitals can obtain allowances for their expenses. There are said to be still 200 auxiliary hospitals unable to obtain the help they need. Yet we hear great complaints from girls who apply of the unnecessary delays which ensue before their services are utilised. The V.A.D. Department at Devonshire House appeals to all trained nurses to assist them in enlisting recruits for the Service. Recognising that nurses in civil hospitals are performing work of great national importance, the authorities are far from wishing to compete for women thus engaged. But they rightly judge that nurses are in a position to influence their friends, often their remote acquaintances, as to the particular kind of war work they ought to choose for themselves or their daughters; they urge nurses, therefore, to suggest to girls who are too young to be trained regularly the existing need for V.A.D. helpers and direct their recruits to communicate with the V.A.D. Department (8), Devonshire House, Piccadilly, W. 1.

The V.A.D. nursing members in naval and military hospitals now receive salaries and allowances of £20 and £30 a year, with board, lodging, laundry, and travelling expenses, and £5 for uniform. This is a satisfactory rate. General service members in these hospitals receive the same rates of pay as in other women's services, an adjustment very desirable for every reason. There can be no doubt that there are special difficulties connected with

**ROUND THE HOSPITALS—(*continued*).**

the organisation of this large body of paid and unpaid workers, but these difficulties are now being faced in the right spirit, and already there is a great change for the better. We are glad to learn that a club for V.A.D. members has been opened at Devonshire House, where three tennis courts will be at their disposal. Adjoining the club-rooms is a canteen for members, and the privilege of using the beautiful gardens of Devonshire House will be warmly appreciated. There is neither entrance fee nor subscription, and the only condition is that members are required to wear correct uniform and hold the uniform permit J.V.A.D. 24.

THE Hon. Sir Arthur Stanley, Chairman of the Joint War Committee, has been appealing for more books for the Red Cross War Library, an agency which now receives only 10 per cent. of the books handed in at the Post Office for the benefit of the troops. This small percentage is not nearly sufficient to provide for the needs of the many hospitals which press for gifts of books. Donors of books and magazines are therefore entreated to send contributions from their surplus store for the use of the sick and wounded to the War Library, Surrey House, Marble Arch, W. 1. Some very fortunate hospitals receive privately an abundant stock of literature, and could easily stimulate their generous friends to help the Central War Library. Visitors to the hospital, for instance, who have constantly under their eyes the cheering effects of light reading on the sick and wounded, might be stirred to begin collecting for this library, and thus a stream of books might be set flowing towards the centre to balance the substantial gifts for ever flowing out to hospitals in all parts of the world.

IT is gratifying to note that in spite of all the troubles at present raging in Ireland, the cause of the nurses is being taken up whole-heartedly, and that an appeal on behalf of the Nation's Fund is being organised throughout the country. An inaugural meeting in connection with the scheme was held at the Royal Hospital, Kilmainham, during the Grand Whitsuntide Fair, when the Marchioness of Waterford presided, and the scheme was outlined by the Hon. Organiser, Mr. Henry McLaughlin. It is intended to raise at least £10,000 by an appeal extending over some four months, and worked simultaneously in all the counties, the work being co-ordinated by an Executive Committee sitting in Dublin, of which Lady Arnott has kindly consented to act as Chairman. As Sir Arthur Stanley was unable to come over for the inaugural meeting, it is intended to hold another public meeting in the second week in June, when he will be present and will be able to give some further explanations about the Nation's Fund. The appeal in Ireland is to be issued under the name of "A Nation's Tribute to Nurses," and in order that no political or party feeling may prevent those who have a genuine admiration for the work of the nurses from participating, it is understood that the money collected will be earmarked for Ireland, and will be applied for the sole purpose of relief of distress amongst certificated Irish nurses. The scheme has already found support amongst all sections of Irishmen, and there is every prospect of its being a great success. It would indeed be a tribute to the nurses if their work in safeguarding the health of the nation should prove a rallying-point of unity in the midst of so much discord and division.

IT is by no means uncommon for recipients of the Second Class of the Royal Red Cross Decoration to be raised subsequently to the First Class. A Royal Warrant now ordains that when this happens the first-named decoration must be returned to the office of the Secretary of State for War.

THE almoner's work at St. George's Hospital, as exposed in the recent report, is carried out with unusual efficiency and completeness. It touches the in-patients no less than the out-patients, and may be said to provide a remarkably comprehensive training in Public Health work for such nurses as are privileged to master its principles. The original aim of the department, to prevent abuse of the charity by persons able to pay for treatment or otherwise ineligible, has been merged in far larger issues, though it is never forgotten. One of the widest purposes consists in "investigation of the home conditions of patients with a view to their permanent improvement." This demands extensive knowledge of State, municipal, and charitable agencies able to co-operate, and could it be fully carried out the submerged tenth would gradually cease to be submerged. Other work done by the almoner is to provide sanatorium and convalescent treatment for those for whom this is advised; surgical instruments, spectacles, artificial teeth, etc., and the supervision of patients with a view to seeing that the clinical treatment recommended is carried out at home. District nurses attend cases requiring dressings or other attention, and the system of after-care tends to ensure the hospital treatment effecting a permanent improvement in the patients' health, instead of, as was often formerly the case, inducing a "hospital habit" and wasting time and money.

GOOD news reaches us of the Elsie Inglis Unit of the Scottish Women's Hospitals, which left London in February after inspection by the King and Queen, and reached its destination on the Macedonian front on April 1st. The camp was constructed forthwith, and consists of tent-wards, laundry, kitchens, incinerator (built of bricks made by the women themselves), and a good sanitary system. Within a fortnight the indefatigable women on the staff were ready for patients. The Jugoslav division of the Serbian Army, to which the Elsie Inglis Unit is attached as sole field hospital, has been in severe fighting ever since then, and the wards are now filled with the sick and

**ROUND THE HOSPITALS**—*(continued)*.

wounded brought straight from the fighting line. The hospital has its own transport, and the girl drivers make daily rounds with the motor ambulances to fetch in the cases. Latest reports speak of the good health and high spirit of all the workers, who have found plenty of obstacles to overcome in the tasks they have assumed, but are triumphing over them with dauntless energy.

---

The Leicester County Nursing Association has made great strides in the past year. The Leicester County Council has voted a sum of 10s. a week for certain necessitous districts, and as a consequence ten new nursing associations have been formed. A second grant from the Local Government Board towards Midwifery and Maternity Service has been distributed to the district nursing associations on the basis of 4s. for each midwifery and 2s. for each maternity case nursed. The National Health Insurance Committee contribute £50 a year as part payment of the salary of the superintendent-nurse, and of establishment charges in return for value received in visiting shelters and nursing tuberculosis cases in the county. Through the principle of co-operation and grants in aid the blessings of the district nursing system are at last becoming a reality through the length and breadth of the land instead of, as formerly, being limited to the favoured spots which could command spontaneous gifts in money and service. Leicester is to be congratulated on the year's work.

---

The amalgamation of the West Sussex Benefit Nursing Association with the Sussex County Nursing Association is a measure which should prove of real advantage to nursing in this county. It was carried through on the recommendation of the Local Government Board to avoid overlapping, and was effected entirely without friction and with practical unanimity. Lady Leconfield, who has rendered great services to the West Sussex Benefit Nursing Association, was elected president of the West Sussex County Nursing Association in its new career of life. The country can no longer afford to allow two persons or two societies to be doing the work of one, and the amalgamation will turn into useful channels much energy now wasted.

---

The new Directory of District Nursing and Streets List for London, issued by the Central Council for District Nursing in London, is a very fine achievement, sufficient in itself to justify the existence of the Central Council, even if this body had not previously been able to show evidence of much valuable work. The form of arrangement is such that this directory cannot, like so many of its kind, become out of date before publication. Its principal feature is a complete list of London streets. By the side of each street name is a reference number, which shows, by means of the index inside the back page of the cover, the name and address of the district nursing association or parish nurse working in the street. The number of nursing associations is thirty, besides the Ranyard nurses, distinguished by the letter "R." In addition, there are fourteen parishes which have trained nurses not attached to any association. These are numbered P 1 to P 14. Thus in all there are forty-five associations and nursing agencies working in the Metropolis. It is a great triumph of organisation, which we owe in the main to the Central Council, that there are no longer districts absolutely unprovided with nursing aid while in others several sets of nurses compete. The directory puts the crowning touch to the long, slow, determined, and inspired work of bringing nursing aid within reach of every house in London, begun nearly half a century ago. At this period of the war, when municipal authorities are fully awake to the value of every citizen's life, it is of priceless value to have this evidence of complete organisation. The directory is indispensable to the almoner's department in every London hospital, and should be in the hands of every social worker. It can be obtained of Messrs. King and Son, Orchard House, Westminster, S.W. 1, price 2s. 6d.

---

Glasgow nurses are to be congratulated on the initial success of the movement recently set on foot by the Scottish Nurses' Association to provide them with a worthy club-house. A small and strictly self-supporting club started last year having proved very successful, it was thought that the time had come for enlisting public support for the purchase and equipment of suitable premises. Mrs. Strong and Mrs. Stewart have consented to act as trustees of the fund, for which a capital of £10,000 is required. Already many handsome sums have been received or promised, and we are glad to find that no one has suggested that the Glasgow nurses need feel any sense of degradation in the reception of this handsome tribute to their services. The new club will, of course, meet its own expenses when once started.

---

The sixth annual general meeting of the Nurses' Insurance Society, the "Separate Section" of the Royal National Pension Fund for Nurses, was held on May 28 at 15 Buckingham Street, Strand. Sir Thomas Dewey, Chairman of the Committee of Management, presided. The amounts paid under sickness and disablement benefit remain on the whole very constant, the total for 1917 being £16,514, as compared with £16,397 in 1916, and £16,338 in 1915. Some 5,000 members of the Fund are on active service. Miss F. A. Cann, R.R.C., Miss S. J. Cockrel, and Miss M. Hardman, who retired by rotation, were re-elected to the committee.

---

☛ It is our invariable practice to pay for all items of news which we may publish. The minimum payment is 5s. Paragraphs of about twenty lines in length—that is to say, of 150 words or less—are preferred. Contributions should be addressed to The Editor, The Hospital, 28 and 29 Southampton Street, Strand, London, W.C. 2, and, to be in time for the current week's issue, should reach him by the first post on Mondays.

☛ For Why Working Nurses Should Articulate, Sir Arthur Stanley's Reply to "Ierne," see p. 185; A National Tribute Fund, p. 186; A Nurse's Charter of Liberty, p. 186.

